# Endocranial Morphology of the Primitive Nodosaurid Dinosaur *Pawpawsaurus campbelli* from the Early Cretaceous of North America

**DOI:** 10.1371/journal.pone.0150845

**Published:** 2016-03-23

**Authors:** Ariana Paulina-Carabajal, Yuong-Nam Lee, Louis L. Jacobs

**Affiliations:** 1 Consejo Nacional de Investigaciones Científicas y Técnicas (CONICET)-Instituto de Investigaciones en Biodiversidad y Medioambiente (INIBIOMA), San Carlos de Bariloche, Argentina; 2 School of Earth and Environmental Sciences, Seoul National University, Seoul, South Korea; 3 Roy M. Huffington Department of Earth Sciences, Southern Methodist University, Dallas, United States of America; University of Lethbridge, CANADA

## Abstract

**Background:**

Ankylosaurs are one of the least explored clades of dinosaurs regarding endocranial anatomy, with few available descriptions of braincase anatomy and even less information on brain and inner ear morphologies. The main goal of this study is to provide a detailed description of the braincase and internal structures of the Early Cretaceous nodosaurid *Pawpawsaurus campbelli*, based on recently made CT scans.

**Methodology/Principal Findings:**

The skull of *Pawpawsaurus* was CT scanned at University of Texas at Austin (UTCT). Three-dimensional models were constructed using Mimics 18.0 (Materialise). The digital data and further processed 3D models revealed inaccessible anatomic structures, allowing a detailed description of the lateral wall of the braincase (obscured by other bones in the articulated skull), and endocranial structures such as the cranial endocast, the most complete inner ear morphology for a nodosaurid, and the interpretation of the airflow system within the nasal cavities.

**Conslusions/Significance:**

The new information on the endocranial morphology of *Pawpawsaurus* adds anatomical data to the poorly understand ankylosaur paleoneurology. The new set of data has potential use not only in taxonomy and phylogeny, but also in paleobiological interpretations based on the relative development of sense organs, such as olfaction, hearing and balance.

## Introduction

Ankylosaurs are a group of quadrupedal armored dinosaurs distributed globally and known from Lower Jurassic to Upper Cretaceous sediments [1–6], and currently divided into two families: the Ankylosauridae and Nodosauridae [3, 6, 7]. The skull in this group is characterized by being highly ossified, with fused or obliterated sutural contacts, particularly in the braincase [6], with few exceptions, such as *Pinacosaurus* [8] and *Kunbarrasaurus* [9]. Moreover, the ventral and lateral aspects of the braincase are often further obscured by the prefrontal and postfrontal laterally, and the pterygoids ventrally. The endocranial morphology is only known for eight taxa. These include the nodosaurids cf. *Polacanthus* sp. from the Early Cretaceous of England [10], *Struthiosaurus transylvanicus* and *S*. *austriacus* from the Late Cretaceous of Austria and Romania respectively [11], *Cedarpelta* from the Early Cretaceous of North America [6, 12], *Panoplosaurus mirus* from the Late Cretaceous of North America [13], *Hungarosaurus* sp. from the Late Cretaceous of Hungary [14] and a partially reconstructed cranial endocast of an unnamed nodosaurid from Japan [15]; whereas *Euoplocephalus* sp. [16, 17] from the Late Cretaceous of North America is the only ankylosaurid studied so far (*Tarchia* and *Talarurus*, from the Late Cretaceous of Mongolia, have been preliminarily studied [18, 19]). More recently, the endocranial morphology of the basal ankylosaur *Kunbarrasaurus ieversi* from the “mid” Cretaceous of Australia was described [9].

The current understanding of the ankylosaurian paleoneurology is, however, still poor. As mentioned, although several cranial endocasts have been described or illustrated, detailed information regarding cranial neurovascular passages, inner ear morphology and airflow system along the nasal cavities is unknown in most taxa [17]. The sources of endocranial information vary. In some cases the endocranial cavity is exposed by fractures, as illustrated by *Talarurus* ([20] see their Fig 7), allowing the direct observation of the structures. In other cases the sediment infilling the cavity is removed mechanically, allowing latex endocasts to be made [12, 14, 21]. More recent studies were made using CT scans, a non-invasive technique [13, 17, 18, 22], that allowed in the best case, digital reconstructions of the ankylosaur cranial endocast, inner ear morphology and nasal cavities. This new set of anatomical information also has potential in paleobiology, as for example olfaction and hearing capabilities were estimated based on the morphology of the sense organs identified in the cranial endocast (see for example [9, 13, 17, 18]). In this sense, the most complete source of information corresponds to those specimens studied using modern techniques and virtual three-dimentional models such as the basal ankylosaur *Kunbarrasaurus*, the nodosaurid *Panoplosaurus* and the ankylosaurid *Euoplocephalus* [9, 13, 17].

We present here a reinterpretation of the braincase neurovasculature and the complete neuroanatomy of the late Early Cretaceous nodosaurid *Pawpawsaurus campbelli* [23] based on X-ray CT scans. The cranial endocast is completely reconstructed including all nerves and vascular elements. In turn, the complete inner ear is the first described for a nodosaurid ankylosaur of such geological age. The nasal cavities are almost completely reconstructed allowing comparisons of the airflow system with other taxa such as *Kunbarrasaurus* [9], *Panoplosaurus* [13] and *Euoplocephalus* [13, 17]. Although the sample of studied taxa is small, the present study provides an interesting opportunity to compare the neurocranial morphology of *Pawpawsaurus* within Nodosauridae and within Ankylosauria, in a relatively wide temporal and phylogenetic range.

## Materials and Methods

**Fig 1:**
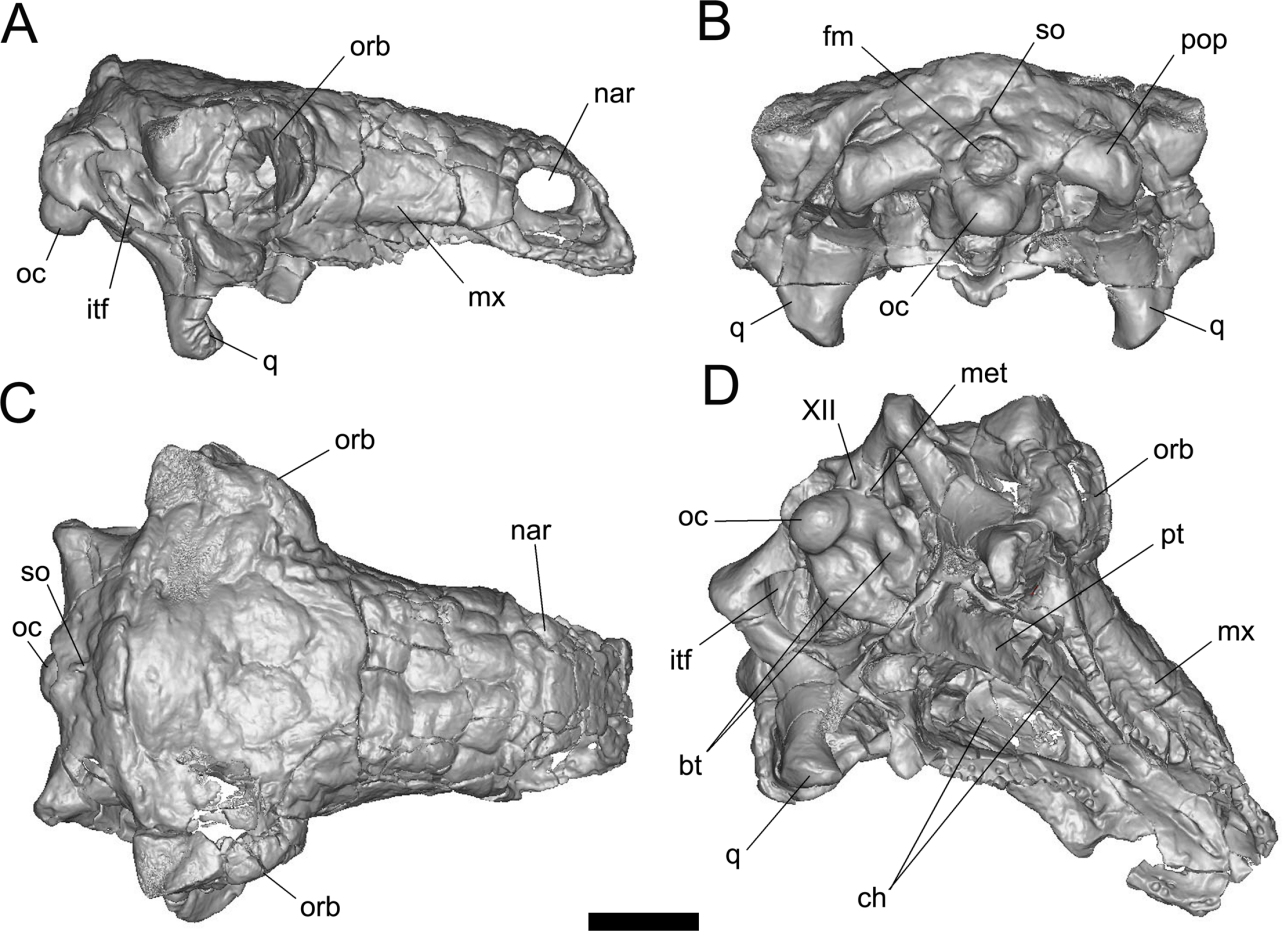
Volume-rendered CT-based reconstruction of the skull of the nodosaur dinosaur *Pawpawsaurus campbelli* (FWMSH93B.00026). Skull in right lateral (A) posterior, (B) dorsal, (C) and lateroventral, (D) views. Abbreviations: bt, basal tuber; ch, choana; fm, foramen magnum; itf, infratemporal fenestra; met, metotic foramen (for CN IX–XI and jugular vein); mx, maxilla; nar, external nostril; oc, occipital condyle; orb, orbit; pop, paroccipital process; pt, pterygoid; q, quadrate; so, supraoccipital. Scale bar equals 5 cm.

The holotype specimen of *Pawpawsaurus campbelli* (described and named with a Southern Methodist University number, SMU 73203, now on display at the Fort Worth Museum of Science and History (FWMSH) inventoried as FWMSH93B.00026) is a complete and well preserved skull (Fig 1). This specimen was found in the upper Albian Paw Paw Formation, approximately 100 Ma [24], Tarrant County, Texas, in 1992. The species was described in detail by Lee [23], who identified most of the external cranial foramina ([23] see their Fig 10). *Pawpawsaurus* was compared to other ankylosaur specimens from the Paw Paw Formation, including a juvenile with a disarticulated basioccipital and basisphenoid [25]; however, only the holotype skull is informative with respect to endocranial morphology.

**Fig 2:**
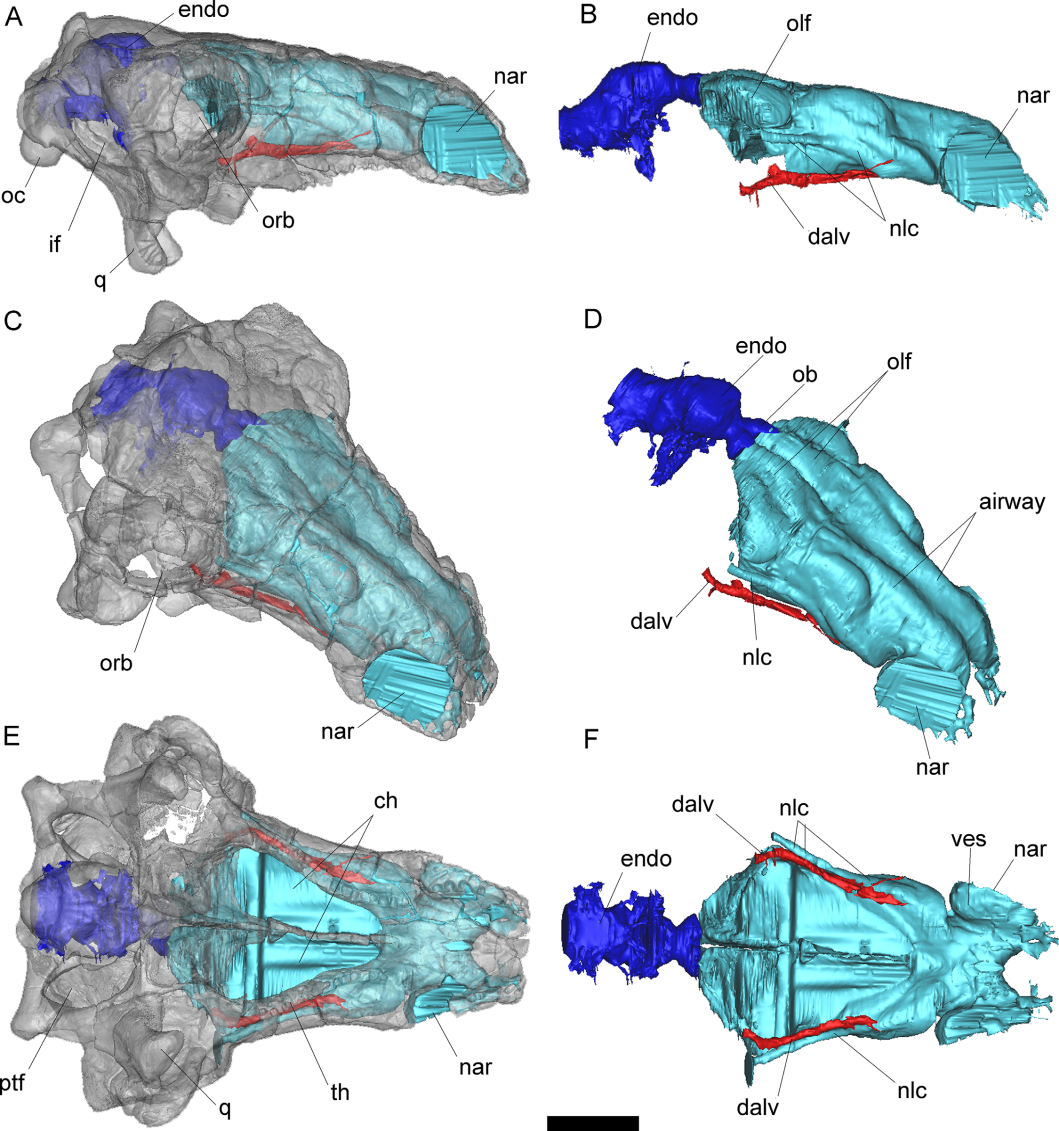
Volume-rendered CT-based reconstruction of the skull of the nodosaur dinosaur *Pawpawsaurus campbelli* (FWMSH93B.00026). In the images of the left side the bone is rendered semitransparent to show the endocranial cavity (in blue) and the nasal cavities (in light blue). Skull right lateral (A,B), right anterolateral (C,D) and ventral (E,F) views. Abbreviations: ch, choana; endo, cranial endocast; if, lateral temporal fenestra; nar, external nostrils; oc, occipital condyle; olf, olfactory region of the nasal cavity; orb, orbit; q, quadrate; th, teeth row; ves, vestibular region of the nasal cavity; 1, dorsal alveolar canal (for maxillary branch of trigeminal nerve, and maxillary vein and artery); 2, nasolacrimal canal (for nasolacrimal duct). Scale bar equals 5 cm.

**Fig 3:**
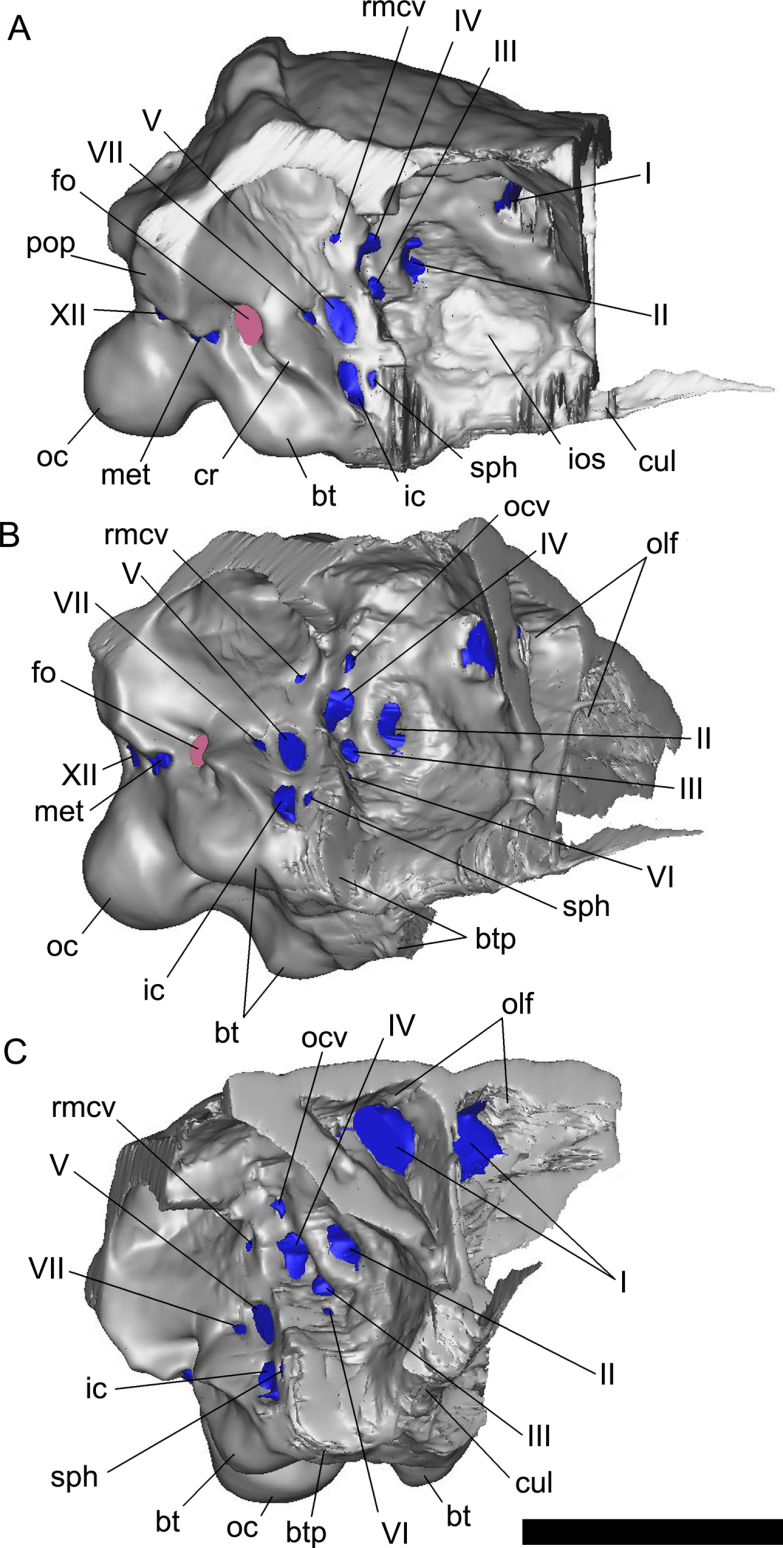
Volume-rendered CT-based reconstruction of the braincase of *Pawpawsaurus campbelli* (FWMSH93B.00026), isolated virtually from other skull bones. Braincase in right lateral (A), lateroventral (B), and anteroventral (C) views. Abbreviations: bt, basal tuber; btp, basipterygoid process; fo, fenestra ovalis; ic, internal carotid artery; ios, interorbital septum; met, metotic foramen (for CN IX–XI and jugular vein); oc, occipital condyle; olf, olfactory region of the nasal cavity; ocv, orbitocerebral vein; pop, paroccipital process; rid, low ridge of bone separating CN II from CN III-IV; rmcv, rostral middle cerebral vein; sph, sphenoid artery; I–XII, cranial nerves. Scale bar equals 5cm.

**Fig 4:**
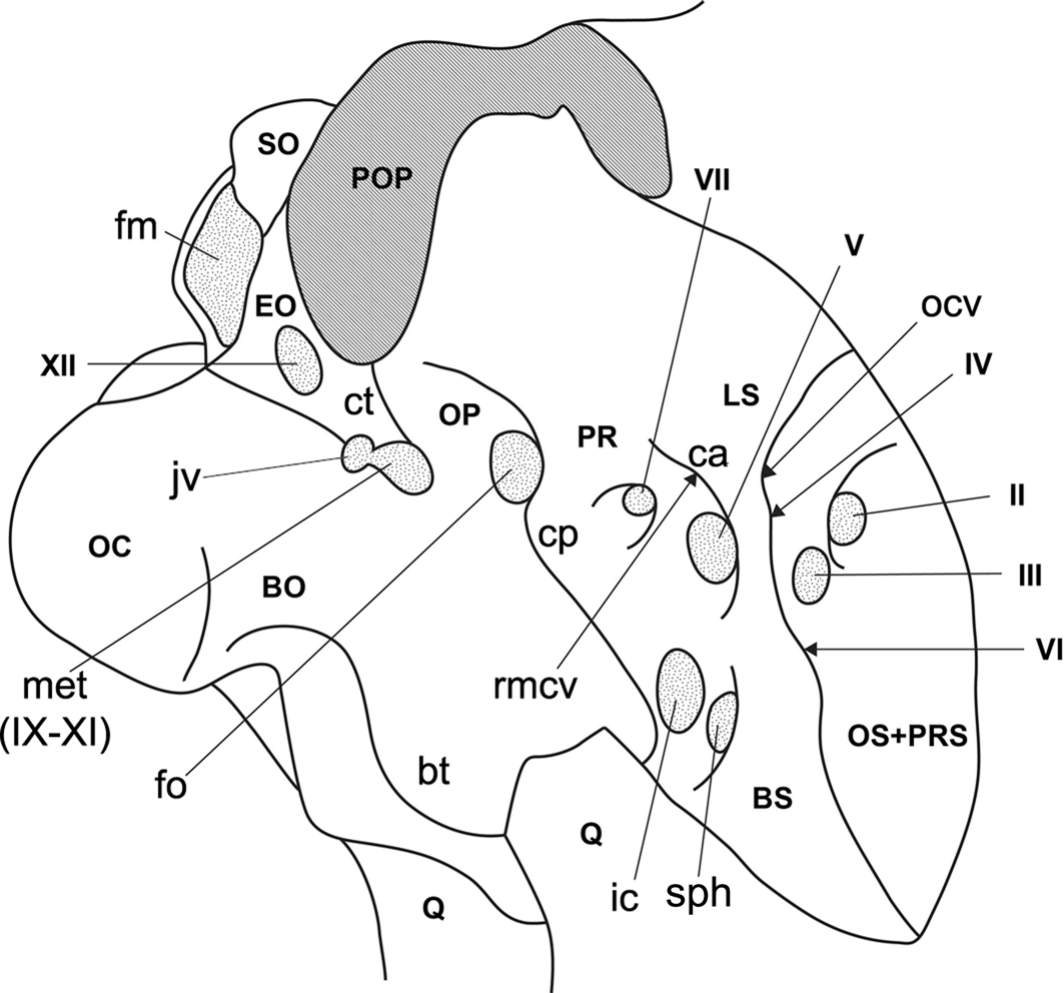
Simplified line drawing of the right lateral side of the braincase of *Pawpawsaurus* (FWMSH93B.00026) showing the re-interpretation of the cranial nerves. Abbreviations: bo, basioccipital; bt, basal tuber; eo, exoccipital; fm, foramen magnum; fo, fenestra ovalis; ic, internal carotid artery; jv, jugular vein; ls, laterosphenoid; met, metotic foramen (for CN IX–XI); oc, occipital condyle; ocv, orbitocerebral vein; op, opisthotic; pop, paroccipital process; pro, prootic; q, quadrate; rmcv, rostral middle cerebral vein; so, supraoccipital; sph, sphenoid artery; II–XII, cranial nerves.

To generate three-dimensional reconstructions of the braincase, cranial endocast, inner ear and nasal cavities, an X-ray CT scan of the skull (FWMSH93B.00026) was performed at the high-resolution X-ray computed tomography facility at the University of Texas at Austin (UTCT), using an NSI (North Star Imaging, Inc.) scanner, with a voltage of 450kV, and a current of 1.5mA. A total of 1815 slices were obtained after a helical continuous CT scan, with an inter-slice spacing of 0.22 mm. Data were output from the scanner in TIF format and then imported into MIMICS 18.0 (Materialise Inc.) for digital extraction of the anatomical features of interest, whereas final illustrations were made with the software Photoshop (CS3) (Fig 2). The braincase was virtually “extracted” to avoid other skull bones that obscured its lateral and ventral views (Fig 3) and a line reconstruction of the lateral wall of the braincase is shown in Fig 4.

The datasets and parameters are archived at www.digimorph.org. (www.DigiMorph.org/specimens/Pawpawsaurus_campbelli/). The 3D PDFs in the Supporting Information (S1 and S2) were generated by exporting the 3D models from MIMICS into MeshLab (Materialise Inc.). The 3D PDFs can be viewed with any computer using the free Adobe Reader program.

**Institutional abbreviations**: **FWMSH**, Fort Worth Museum of Science and History, Fort Worth; **MCM**, Mikasa City Museum, Hokkaido, Japan; **MMCH**, Museo “Ernesto Bachmann”, Villa El Chocón, Argentina; **YPM**, Yale Peabody Museum, New Haven; **USNM**, National Museum of Natural History, *Smithsonian Institution*, Washington, USA; **ROM**, Royal Ontario Museum, Toronto, Canada.

## Description

### Cranial endocast

**Fig 5:**
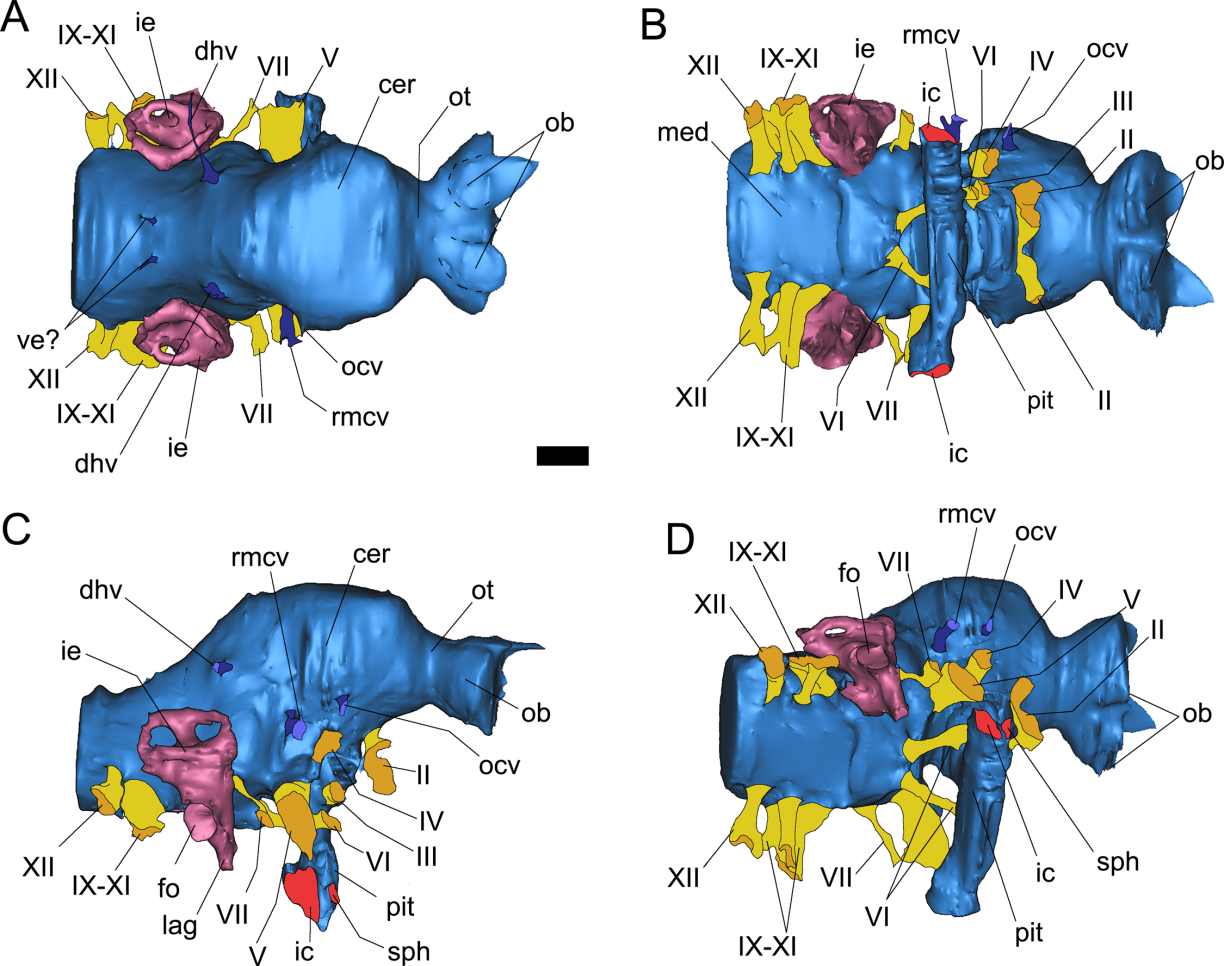
Surface-rendered CT-based reconstructions of the cranial endocast and left endosseous labyrinth of *Pawpawsaurus campbelli* (FWMSH93B.00026). In the figures, the right inner ear is a mirrored image from the left inner ear. Cranial endocast in dorsal (A), ventral (B), right lateral (C), and lateroventral (D) views. Abbreviations: cer, cerebral hemisphere; dhv, dorsal head vein; fo, fenestra ovalis; ic, internal carotid artery; ie, inner ear; lag, lagena; med, medulla; ob, olfactory bulb; ocv, orbitocerebral vein; ot, olfactory tract; pit, pituitary;; rmcv, rostral middle cerebral vein; sph. sphenoid artery; I–XII, Cranial nerves; ve? vein?. Scale bar equals 10 mm.

The endocranial cast of *Pawpawsaurus* is completely reconstructed providing a general image of the superficial topography of the brain (Fig 5). It reveals the main structures and proportions of hindbrain, midbrain, and forebrain [21, 26, 27]. The morphology of the cranial endocast of *Pawpawsaurus* is, in general, similar to that described for other ankylosaurs [9–11, 13–17], being anteroposteriorly short and having a globose forebrain and enlarged internal carotid arteries that transversely enter the distal end of the pituitary fossa (except in *Kunbarrasaurus* [9]). The cranial endocast represents approximately 30% of the skull length; whereas the same ratio is 25% in *Euoplocephalus* and 40% in *Kunbarrasaurus* [9]. In *Panoplosaurus* ([13] see their Fig 7C) the length of the brain represents 33% of the skull length, suggesting a ratio of approximately 30% for nodosaurids, higher than that of derived ankylosaurids. The endocast of *Pawpawsaurus* is approximately 96 mm long from the foramen magnum to the olfactory bulbs, and has a maximum width of 35 mm across the cerebral hemispheres. The olfactory tract is relatively short and the space occupied by each olfactory bulb is oval-shaped and 13.5 mm long. The general shape of the cranial endocast in lateral view (Fig 5C) resembles that of cf. *Polacanthus* ([14] see their Fig 6.1).

#### Forebrain

The features of the forebrain observed in the cranial endocast of *Pawpawsaurus* include the olfactory tracts and olfactory bulbs (CN, cranial nerve I), the cerebral hemispheres, the optic nerves (CN II), and the pituitary body.

The olfactory tract is 5 mm long and 16.8 mm wide, and slightly ventrally oriented. In the nodosaurids *Panoplosaurus* and *Hungarosaurus*, the olfactory tract and bulbs incline slightly ventrally, whereas in *Struthiosaurus* the olfactory bulbs are markedly ventrally oriented ([14] see their Fig 6). In *Pawpawsaurus*, the cavities occupied by the olfactory bulbs are oval-shaped and divergent from the midline at an angle of approximately 75°, similar to the condition described for the nodosaurid *Panoplosaurus (*[13] see their Fig 7D) and the ankylosaurid *Euoplocephalus* [16] and ([13] see their Fig 8D).

Recent studies state that in non-avian and avian theropods, the olfactory ratio [28] is a measure of the relative importance of olfaction through large scale evolution, where larger ratios indicate better olfactory capabilities (the olfactory ratio is calculated as the ratio between the longest diameter of the olfactory bulb and the longest diameter of the cerebral hemisphere, regardless of orientation, multiplied by 100). The olfactory ratios in *Pawpawsaurus* (46.2), *Panoplosaurus* (44.0, based on [13], see their Fig 7), and *Euoplocephalus* (52.0, based on [13], see their Fig 8) are approximately similar, suggesting a similar olfaction capability for the three taxa (the ankylosaurid taxon showing the largest ratio). The olfactory ratios calculated here for the three mentioned ankylosaurs are similar to those ratios calculated for ceratosaur theropods, and the extant crocodile *Alligator* (see [28] and their Table 1].

The cerebral region is domed dorsally, although in lateral view the dorsal margin appears more flattened (Fig 5C). In dorsal view the fissura interhemispherica is not visible, indicating the presence of an enlarged dorsal longitudinal venous sinus and a thick dura mater, as in most other dinosaurs [21, 29], but unlike derived non-avian theropods and hadrosaurs [30, 31]. However, the cerebral hemispheres are easily discernible in the endocast. They are dorsally and laterally expanded, as in other ankylosaurs [13, 14]. In lateral view, the cerebrum is slightly elevated above the level of the cerebellum, as in *Panoplosaurus*, *Struthiosaurus* and *Hungarosaurus*, but unlike the markedly dorsally expanded cerebrum in cf. *Polacanthosaurus* [14].

The complete cast of the pituitary chamber could not be reconstructed. However, the general shape and orientation is discernible in the CT scans, showing that the pituitary descends vertically from the ventral surface of the endocast. The infundibular stalk is anteroposteriorly short and transversely wide (Fig 5B–5D). The pituitary is distally confluent with the two large passages for the internal carotid arteries, which enter the pituitary cavity transversely, at an angle of 180°, as in other ankylosaurs [17–19] (Fig 4B and 4C), except *Kunbarrasaurus* [9]. On the lateral side of the basicranium, a small vascular foramen, probably for the sphenoid artery [17, 32, 33] lies just anterior to the internal carotid artery, entering the basisphenoid and joining the internal carotid artery passage (Fig 5C and 5D).

The optic nerves (CN II) have short passages, divergent from the midline (Fig 5B) and each exit the braincase through large and oval foramina, probably enclosed only by the orbitosphenoids. On the lateral wall of the braincase, the foramen for CN II is separated from CN III and IV by a curved shallow ridge of bone (Fig 3).

#### Midbrain

The visible mesencephalic structures in the endocast of most dinosaurs consist of the optic lobes and cranial nerves III and IV. The optic lobes in *Pawpawsaurus* are not clearly defined in the endocast (Fig 5). As in *Panoplosaurus* [13] the dorsal surface of the endocast is smooth and no “epiphysis marks” are observed as in *Struthiosaurus* and *Hungarosaurus* [14].

On the lateral wall of the braincase, immediately posterior to the foramen for CN II, there are three large foramina vertically disposed, and separated from the optic nerve by a shallow ridge of bone anteriorly (Fig 3B). The dorsalmost foramen is the smallest in diameter, and transmitted the orbitocerebral vein, which is found for example in other ankylosaurians [9, 17, 29] as well as in other dinosaurs such as sauropods [34–36], ceratopsians [33] and theropods [37, 38]. The large foramen located below the orbitocerebral vein corresponds to the CN IV (Fig 3). Due to the quality of the CT scans it is unclear whether a passage confluent with the proximal section of the infundibular area (identified in *Euoplocephalus* as the “sinus of the pituitary” [17]) occurs. The larger foramen ventral to CN IV and posteroventral CN II corresponds to CN III (Figs 3 and 4). Within the studied ankylosaurs, separate foramina for CN III and IV were described in *Cedarpelta* [12]; whereas in *Euoplocephalus* the roots for CN III and IV share the same passage exiting the braincase though a single foramen [16,17] (contra [39] which illustrate separate foramina for this taxon), as in the basal ankylosaur *Kunbarrasaurus ieversi* [9]. Our reinterpretation of *Pawpawsaurus* cranial foramina III–IV based on the CT scans is shown in Fig 4.

#### Hindbrain

The visible features in this region of the cranial endocast include the cerebellum, the medulla oblongata, and cranial nerves V–XII (CN VIII is not observed in the CT scans). The cerebellar region is not expanded dorsally but slightly laterally. The development of the cerebellar area in cranial endocasts in ankylosaurs has been related to locomotor capabilities [14]. However, in *Pawpawsaurus* most of the lateral expansion forming a well-marked vertical bulge- is probably not due to an expansion of the cerebellum itself, but to an enlarged transverse venous sinus (Fig 5C). An enlarged vascular sinus running ventrally from the dorsal longitudinal sinus (see for example [29]) to reach the trigeminal nerve was identified as the infilling of the dorsal head/caudal middle cerebral vein system in the sauropod *Spinophorosaurus* [35]. This sinus is also evident in other basal sauropods such as dicraeosaurids [40] and rebbachisaurids [41, 42], although not well developed in theropod and ornitischian dinosaurs. The transverse sinus present in *Pawpawsaurus* and other ankylosaurs (cf. *Polacanthus* and *Panoplosaurus* [14], see their Fig 6.1, 6.6]) is relatively larger than that observed in sauropods. In the endocasts of *Struthiosaurus*, *Hungarosaurus* and cf. *Polacanthus* ([14] see their Fig 6), the dorsal longitudinal sinus is better developed than in *Pawpawsaurus*. In *Pawpawsaurus*, there is no floccular recess on the wall of the vestibular eminence as in other nodosaurids, but unlike some ankylosaurids [17, 18].

The medulla oblongata is anteroposteriorly short and transversely wide (Fig 5B). The width of the medulla is similar to the width of the cerebral region (Fig 5A). In lateral view, the ventral margin of the medulla oblongata is flat (Fig 5B), as in cf. *Polacanthus* and *Struthiosaurus*, but unlike the ventrally concave medulla observed in *Hungarosaurus* and *Panoplosaurus* ([14] see their Fig 6.5, 6.6).

All branches of CN V exit the braincase through a single foramen, located dorsal to the internal carotid foramen, at the level of the base of the basipterygoid process (Figs 3A and 5C and 5D). On the lateral side of the braincase, a well-defined vertical ridge separates CN V from CNs III and IV (Fig 3C). The laterosphenoid and the prootic, probably form the margins of the trigeminal foramen, as in most dinosaurs [43] and extant archosaurs [44]. The passage for CN V is large in diameter (approximately 7 mm), and runs slightly ventrolaterally from the ventral section of the endocast (Fig 5C).

The CT scans provided new information that allowed us to identify the small, anteriorly directed foramen for CN VI, ventral to CN III, which is not observed in lateral view of the braincase in the articulated skull (Fig 3B). Each root for CN VI is close to the midline and its counterpart, and each passage is anteroventrally oriented, diverging from the middle at an angle of approximately 55° (Fig 5B and 5D). The passage is small in diameter and runs lateral to the pituitary, exiting the braincase through a small foramen located ventral to CN III (Fig 3C).

The passage for CN VII is small in diameter compared with CNs II–V. It is long and anteroventrally projected, exiting the braincase though a small foramen just posterior to CN V (Fig 3A). On the lateral side of the braincase, CNs V and VI open within a shallow depression delimited by the crista antotica anteriorly, and by the crista prootica posteriorly (Fig 3C).

The passage for CN VIII is poorly visible in the CT scans and is not shown in the digital endocast. The small-diameter passage, running from the endocranial cavity into the vestibular area of the inner ear, may represent one of the branches of the auditory nerve.

Cranial nerves IX–XI and the jugular vein exit the braincase through the metotic foramen, which is clearly visible in posterior view and partially visible in lateral view (Figs 1D and 3B). The foramen is 8-shaped due a constriction that is separating a larger lobe anteriorly and a smaller lobe posteriorly, the latter probably for the jugular vein [23]. Although separate branches for the different nerves are not clearly visible in the CT scans, there is a separate passage (smaller in diameter) running slightly posteroventral to the main passage for CNs IX and XI, and joining it before exiting the braincase through the posterior section of the metotic foramen (Fig 4). This passage may represent a separate canal for the anterior branch of CN XII, which is merged to the posteroventral corner of the metotic foramen in *Euoplocephalus* [17].

In *Pawpawsaurus*, the quality of the CT scans allowed the recognition of a single passage for all the branches of CN XII (Fig 1B). However, separate roots for the hypoglossal nerve branches may have joined before exiting through the single foramen on the lateral side of the braincase (Fig 5B–5D). Furthermore, the elongate shape of the reconstructed root suggests the presence of two adjacent branches, one dorsal and one ventral. The nodosaurid *Polacanthus* [10] and the ankylosaurids *Euoplocephalus* sp. and *Struthiosaurus* ([14] see their Fig 6) have two passages for the branches of the hypoglossal nerve.

#### Vascular elements

The internal carotid artery, orbitocerebral vein, rostral middle cerebral vein, and the caudal middle cerebral vein are identified in the endocast. As mentioned, the large external foramen for the internal carotid artery is located on the basicranium ventral to CN V and enclosed by the parabasisphenoid (Fig 3A). The internal carotid artery passage has a large diameter and enters transversely the distal end of the pituitary (Fig 5B). The large size and the mostly transverse disposition of the internal carotid passage is also observed in the ankylosaurids *Euoplocephalus* [17], *Tarchia* and *Talarurus* [18, 19], indicating that this character is plesiomorphic for Ankylosauria.

In the braincase, adjacent to the internal carotid artery foramen is the foramen for the sphenoid artery, as described in *Euoplocephalus* [17] (Figs 3A and 5C). In *Pawpawsaurus*, however, the passage for this vascular element joins the passage of the internal carotid artery on its way into the pituitary fossa, instead of entering the pituitary fossa separately. Foramina in the basisphenoid communicating with the pituitary cavity are present in *Talarurus*, identified as the “pituitary vein” and the “ophthalmic artery” by Turmanova ([20] see their Fig 7A); whereas Norman and Fraiers [10] described a combined passage for the “palatine artery” in cf. *Polacanthus*. In other dinosaurs, this foramen has been identified as for the “sphenoid artery” in titanosaurid sauropods (e.g. [45]; “pituitary vein” in [34]; the ceratopsid *Pachyrhinosaurus* [33]; and the abelisaurid theropod *Majungasaurus* [37]). We follow here the terminology used by the authors mentioned above [33, 35], who interpret the foramina leading into the pituitary fossa as the foramen for the sphenoidal artery, as present in birds [32].

As mentioned, the CT scans allowed the identification and description in *Pawpawsaurus* of the orbitocerebral vein, the rostal middle cerebral vein, and the dorsal head vein for the first time. The orbitocerebral vein [29] has a small passage ventral to the cerebral hemisphere that runs laterally to exit the braincase into the orbital vault area (Fig 3D) through a small foramen located dorsal to CN IV (Fig 5C and 5D). The cerebral branches of the dorsal longitudinal sinus drain into the orbit via the orbitocerebral vein canals [29, 33]. An orbitocerebral vein was identified in *Kunbarrasaurus* [9] and *Euoplocephalus* [17], whereas a similarly located blood vessel was identified as the “vena capitis anterior” in the Hokkaido nodosaurid [15] and as the “vena cerebralis media” in *Polacanthus* [10]. When this foramen is not present, the vein drains through the CN IV foramen [29, 40, 46].

The rostral middle cerebral vein has a short passage located dorsal to the root of the trigeminal nerve (CN V) (Fig 5C and 5D). The external foramen for this vein is located posterior to the orbitocerebral vein and separated from it by a robust ridge of bone (Fig 3A). A shallow vertical ridge on the endocast joins the rostral middle cerebral vein with the passage for CN V, indicating the presence of a transverse venous sinus, as observed in other dinosaurs such as titanosaurs [34]. The dorsal head vein is dorsal to CNs V and VII, and is widely separated from its counterpart in dorsal view (Fig 5A). Its passage is small in diameter and is partially reconstructed on the left side. It runs from the dorsal expansion (dural peak) of the endocast, slightly laterodorsally to exit into the dorsal and median section of the supratemporal fossa. The exit foramen for this vein is obscured by sediment and is not visible in the lateral view of the braincase. A pair of small passages lies on the dorsal margin of the medulla, close to the midline. Only the base of these passages can be reconstructed using CT scans. These are probably associated with the dorsal venous longitudinal sinus.

The orbitocerebral vein, the rostral middle cerebral vein and the dorsal head vein have been described in the ankylosaurid *Euoplocephalus* (“posterior middle cerebral vein” in [17] and the basal ankylosaur *Kunbarrusaurus* [9]). In the nodosaurid *Polacanthus* [10] four vessels are recognized on the endocast as the “vena cerebralis media”, one located dorsal to the cerebellum, one located dorsal to CN V, and two anterodorsal to CN IV. Based on their relative positions with respect CNs V and IV, they probably correspond to the dorsal head vein, cerebral vein, rostral middle cerebral vein, and orbitocerebral veins, respectively.

### Inner ear

**Fig 6:**
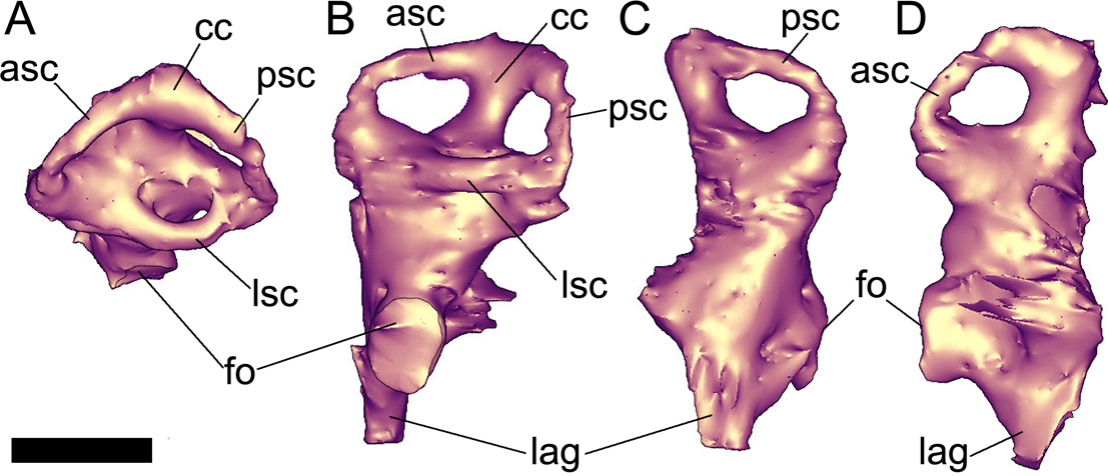
Digital reconstruction of the left inner ear of *Pawpawsaurus campbelli* (FWMSH93B.00026). In dorsal (A), lateral (B), posterior (C), and anterior (D) views. Abbreviations: asc, anterior semicircular canal; cc, crus communis; fo, fenestra ovalis; lag, lagena; lsc, lateral semicircular canal; psc, posterior semicircular canal. Scale bar equals 10 mm.

The left inner ear of *Pawpawsaurus* was digitally reconstructed (Fig 6), whereas the right inner ear is a mirrored image, as presented in Fig 5. The general morphology of the inner ear is similar to that described in other ankylosaurs [13,16–18], except in *Kunbarrasaurus* [9] in which the semicircular canals are short, robust, and low, with a robust, conical and elongate lagena. Although a preliminary study suggested the presence of an elongate lagena in *Pawpawsaurus* [18], further identification of the fenestra ovalis indicates that the lagena is in fact short (approximately 5 mm length).

In *Pawpawsaurus*, the complete inner ear is approximately 27 mm tall and has a maximum width of 15.5 mm at the level of the semicircular canals (Fig 6). The anterior semicircular canal is slightly larger than the posterior semicircular canal, although the dorsal margins of both canals are approximately at the same level, unlike the condition in *Euoplocephalus* [13] in which the anterior semicircular canal is larger than the posterior canal. In *Pawpawsaurus*, the angle formed between the anterior and posterior semicircular canals is approximately 100° (Fig 6A). The lateral semicircular canal is robust, with a markedly small diameter (Fig 6A and 6B).

The fenestra ovalis marks the limit between the vestibulum and the lagena, indicating that the length of the lagena in *Pawpawsaurus* is shorter than the semicircular canals in lateral view (Fig 6). Similar relative size of the lagena is observed in most basal theropods (e.g., [37], see their Fig 19 and [47], see their Fig 8U), sauropods (e.g., [41], see their Fig 1G and [34], see their Fig 9) and ornithischians (e.g., [33], see their Fig 3 and [48], see their Fig 8). However, the lagena in *Euoplocephalus* is clearly as long as or longer than labyrinth height [13, 17], and this seems to be true also for the Mongolian genus *Tarchia* [19]. Miyashita et al. [17] stated that hearing was probably the most important sense for ankylosaurs. The shorter lagena observed in *Pawpawsaurus* suggests that hearing was probably an important sense in ankylosaurids [17] but less so in nodosaurids and probably the basal ankylosaur *Kunbarrasaurus* [9].

### Nasal cavity

Each paired nasal cavity comprises a tube that opens outside through the external nares and to the mouth trough the choanae. As in other vertebrates, three main parts are recognized within the nasal cavity: an anterior vestibulum, a middle cavum proprium (respiratory and olfactory conchae lie within this section), and a postseroventral nasopharyngeal duct [49–51]. In lateral view, the cast of the nasal cavity is oriented at an angle of approximately 155°–160° with respect to the main axis of the endocast (Fig 2B), similar to that observed in *Kunbarrusarus* [9], whereas the angle is approximately 135°–140° in *Panoplosaurus* ([13] see their Fig 7E), and 120° in *Euoplocephalus* ([13] see their Fig 8C).

**Fig 7:**
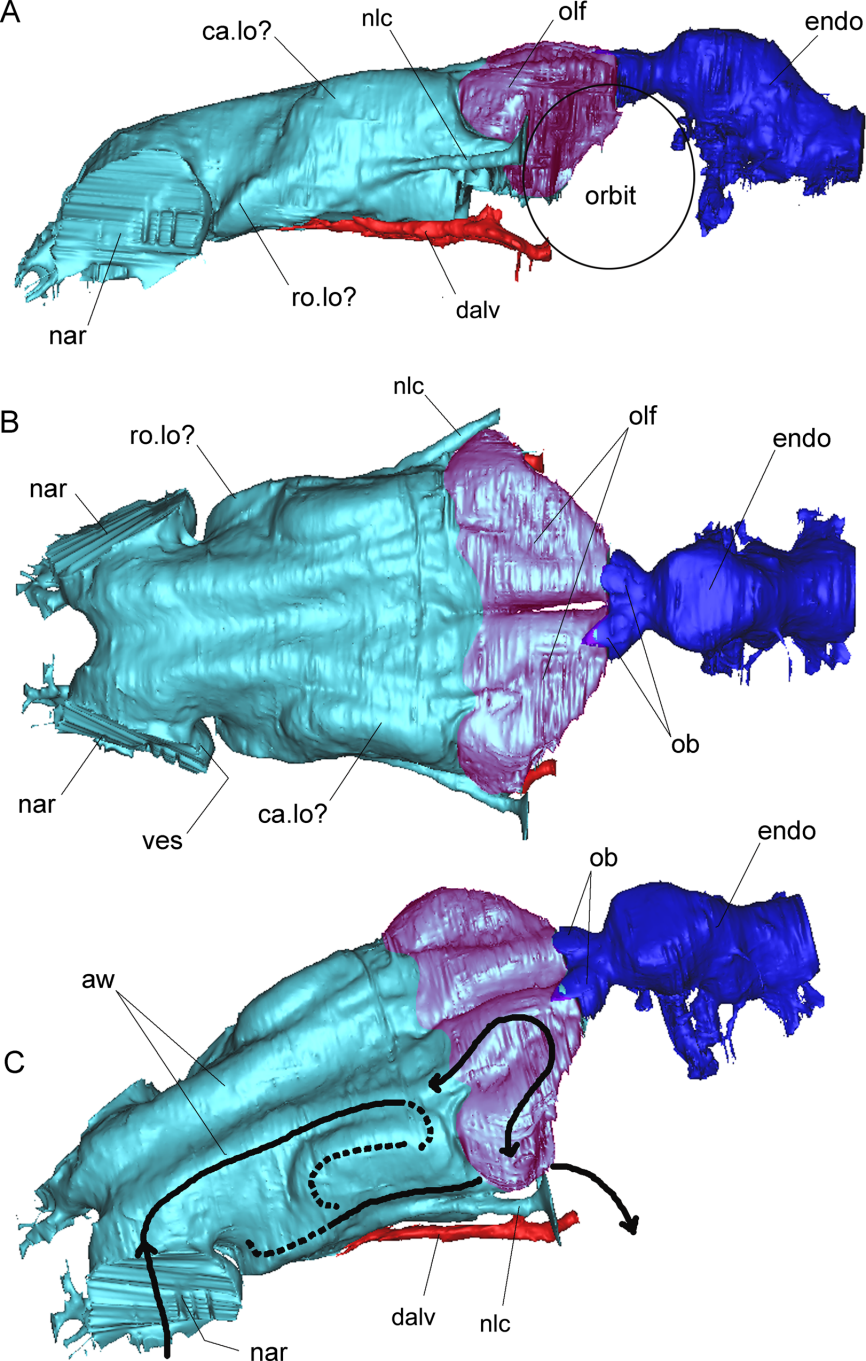
Surface-rendered CT-based reconstruction of the cranial endocast and nasal cavities of *Pawpawsaurus campbelli* (FWMSH93B.00026). The posterior olfactory region of the nasal cavity is indicated in color magenta. The airflow pathway interpretation is indicated as black arrows in (C). In left lateral (A), dorsal (B) and lateroventral (C) views. Abbreviations: aw, airway; ca.lo, caudal loop?; dalv, dorsal alveolar canal (for maxillary branch of trigeminal nerve, and maxillary vein and artery); endo, endocranial cavity; nar, external nostril; nlc, nasolacrimal canal (for nasolacrimal duct); ob, olfactory bulbs; olf, olfactory region; ro.lo, rostral loop?; ves, vestibulum of the nasal cavity. Scale bar equals 10 mm.

The nasal cavity of *Pawpawsaurus* is complete, although the internal laminae delimiting the loops of the airway described in *Panoplosaurus* and *Euoplocepahlus* [13, 17] are not preserved, probably because they were cartilaginous (Figs 2 and 7). The CT scan shows on the left side (the unprepared side of the braincase) a series of low ridges partially segregating the various cavities, which probably represent incomplete or unossified laminae. The impression of those loops of the airway on the internal walls of the nasal cavity allow a partial three-dimensional reconstruction of the dorsal morphology of the airflow loopings (Fig 7A and 7B). The interpretation of the airway path inside the nasal cavity and airway of *Pawpawsaurus* (Fig 7C) is consistent with the morphology and airway path described for *Panoplosaurus* and *Euoplocephalus* [13,17]. In both these Late Cretaceous forms, the airflow ascends from the nostril caudodorsally forming the rostral loop, turns laterally, then rostroventromedially completing the loop below the ascending tract, and then ascends again caudodorsally forming a second loop which arches caudoventrally into the choana [13].

In *Pawpawsaurus*, the nasal cavities are separated by a median ridge, although this separation is complete only posteriorly (Fig 7B). In dorsal view, there are two main sectors in each nasal cavity. The posterior part is adjacent to the olfactory bulb and corresponds to the olfactory region of the nasal cavity, as interpreted by Witmer and Ridgely [13] in *Panoplosaurus* and *Euoplocephalus*. Each olfactory region is in turn subdivided by a deep longitudinal groove, resulting in what seems to be a posterior looping of the airway path near the olfactory bulb. This part of the cavity offers the surface area for the olfactory epithelium [48, 52]. In *Pawpawsaurus*, the olfactory region of each side is separated by a longitudinal groove, suggesting the presence of a third loop in the airflow, although the flow direction is uncertain (Figs 2D and 7). A similar separation of the olfactory region is present in *Euoplocephalus*, where the median part corresponds to the caudal loop of the airway path ([13] see their Fig 8B) and the lateral expansion corresponds to the olfactory cavity.

The anterior section of the nasal cavity is anteroposteriorly elongate and represents the rostral and caudal loops of the airway path described in *Panoplosaurus* [13]. In *Pawpawsaurus* these loops are not markedly expanded dorsally (Fig 7). As observed in dorsal view, each naris leads into an anteroposteriorly elongate tube. The airflow then turns laterally and then anteroventrally forming the anterior loop, similar to but less laterally pronounced than in *Panoplosaurus*(Figs 2B and 7).The caudal loop however, is not clearly discernible, but seems to lie in a vertical plane (Fig 7), whereas both, rostral and caudal loops are horizontal in *Panoplosaurus*.

**Fig 8:**
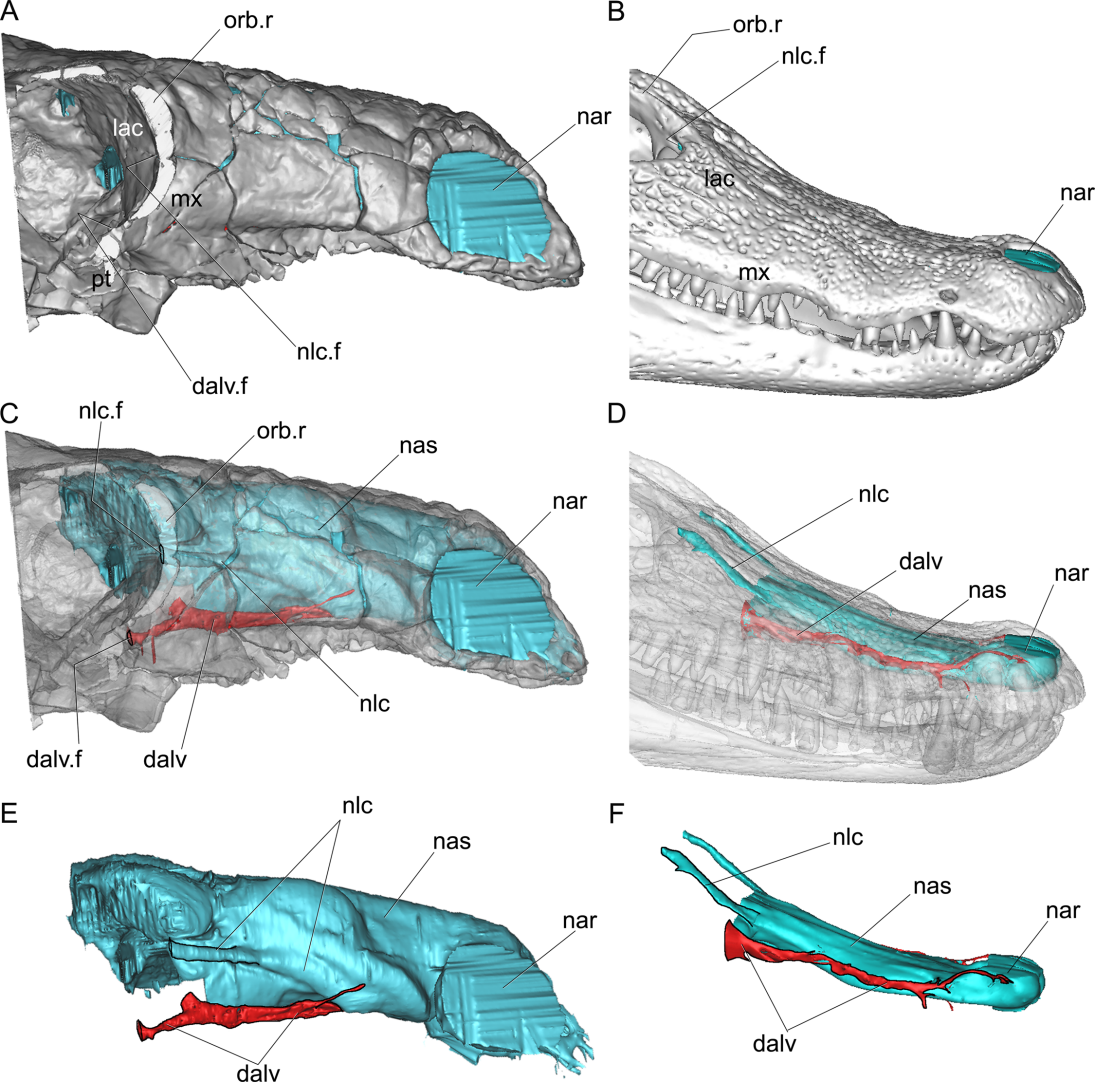
Detail of the skulls of *Pawpawsaurus campbelli* (FWMSH93B.00026) (left) and the extant crocodile *Caiman* (right) in right lateral view. In the images at the top the bone is rendered solid (A,D), whereas in the images below, the bone is rendered semitransparent to show the nasal cavity and the neurovascular passages (B,E). In *Pawpawsaurus*, the lateral section of the orbital margins was sectioned parasagittaly to allow the observation of the anterior wall of the orbit. Nasal cavity and neurovascular passages rendered isolate (C,F). Abbreviations: dalv, dorsal alveolar canal (for maxillary branch of trigeminal nerve, and maxillary vein and artery); dalv.f, dorsal alveolar canal foramen in the anterior wall of the orbit; lac, lacrimal; mx, maxilla; nar, external nostrils; nas, nasal cavity; nlc, nasolacrimal canal; nlc.f, nasolacrimal canal/duct foramen; orb.r, orbital rim; pt, pterygoid. Not to scale.

In *Pawpawsaurus*, two large parallel neurovascular passages related to the nasal cavity lie on each side of the skull (Fig 2B). Both passages open on the anterior wall of the orbit (Figs 2B and 8) and communicate anteriorly with the nasal cavity. The ventral passage runs along the maxilla (Figs 2, 6 and 8) and corresponds to the dorsal (= superior) alveolar canal, as the one observed in extant reptiles, such as crocodiles ([53] see their Fig 3a). In extant reptiles [54] the maxillary branch of the trigeminal nerve (n. alveolaris dorsalis caudalis [52, 55] or “superior alveolar nerve”, for innervation of the teeth, surface of the snout and inner surface of the nasal passage [56]), plus vascular elements such as the maxillary artery and vein [54, 57] (e.g. infraorbital artery in [56, 58]), passed through this canal. In *Pawpawsaurus*, it runs parallel to the tooth row, indicating its relation with the maxillary branch of the trigeminal nerve [57]. The dorsal alveolar canal has an external foramen located ventrally on the anterior wall of the orbit (”superior alveolar foramen” in [57], “infraorbital foramen within the maxilla” in [54]), which is enclosed probably between the fused pterygoid and the maxilla in *Pawpawsaurus* (Fig 8).

The dorsal passage corresponds to the nasolacrimal canal, for the nasolacrimal duct. It is adjacent to and ventrally confluent with the airway path below the rostal loop (Figs 2,6 and 8), having an external foramen located dorsally on the anterior wall of the orbit (Fig 8). In crocodilians, the nasolacrimal duct passes through the lacrimal bone from the orbit to the nasal cavity, draining the lacrimal fluid [52, 55]. In *Pawpawsaurus*, as in most ankylosaurs, skull sutures are obscured by fusion and it is not possible to establish which bones form the anterior wall of the orbit. In *Pinacosaurus* however, the participation of the lacrimal in the rostral margin of the orbit was described [59] together with a large “lacrimal foramen”. The nasolacrimal duct was also recently described within the lacrimal of *Kunbarrasaurus* [9]. In *Pawpawsaurus*, the location of this foramen and canal (running across the lacrimal), and its relation with the nasal cavity, supports its identification as the nasolacrimal canal (Fig 8).

## Comments on Paleobiological Implications of Endocranial Morphology

### Nasal cavities and airway path

Olfaction and vomeronasal chemoreception constitute the chemical senses associated with the nasal region, which play an important role in sensory, thermal, and respiratory physiology [50, 51]. In ankylosaurs, the nasal cavity has a rich blood supply, as described for *Euoplocephalus* [13, 17], *Panoplosaurus* [13], and *Pawpawsaurus* in the present study. In *Pawpawsaurus*, the olfactory region of the nasal cavity is anteroposteriorly shorter than the cranial endocast, whereas in Late Cretaceous taxa the olfactory part of the nasal cavity is as long as the endocranial cast.

In ankylosaurs the external nares are delimited by deep depressions [60]. This indicates a deep vestibule, which is clearly differentiated in the endocast from the main nasal cavity (Figs 2F and 7B). In this anterior region of the nasal cavity the external nasal gland is located. It is usually the largest of the nasal glands in all orders of extant Reptilia, and it is located dorsally and laterally within the nasal cavity, between the cartilaginous nasal capsule and the bony skull [49]. Osmólska [60] stated that extended bony nostrils present in ankylosaurs are common in large herbivorous dinosaurs and indicate the presence of a salt nasal gland. However, in other extant reptiles such as some lizards (e.g., iguanids) the elongation of the vestibulum of the nasal cavity is correlated with a specialization for desert life [61] by preventing entrance of sand particles. In the cast of the nasal cavity of *Pawpawsaurus*, there is no evidence of the presence of an enlarged nasal gland, nor the presence of a salt gland. Although the anterior region of the nasal cavity may have hosted an enlarged nasal gland, the function of the enlarged vestibulum in *Pawpawsaurus* and other ankylosaurs remains unknown.

The airway paths in *Pawpawsaurus* (Fig 7C) and *Panoplosaurus* are simpler in shape and size compared to the complex airway path developed in *Euoplocephalus* [13]. In *Pawpawsaurus*, the rostral and caudal loops seem to be more vertical and less laterally expanded than in *Panoplosaurus*. Witmer and Ridgely [13] concluded that the nasal airway of nodosaurids (i.e., *Panoplosaurus*) was more complicated than previously thought, although it seems simple when compared to the nasal airway of ankylosaurids (i.e., *Euoplocephalus*). In turn, the airway path in the nasal cavity of *Pawpawsaurus* seems to be similar to its more derived relative *Panoplosaurus*, although some differences can be noted (e.g. relative size of endocranial cast/nasal cavity; the angle between the cranial endocast and the nasal cavity is wider in *Panoplosaurus*; the rostral and caudal loops are disposed more vertically in *Panoplosaurus* and they are more dorsally expanded than in *Pawpawsaurus*) suggesting an enlargement and lateralization of the anterior and posterior loops in the Late Cretaceous form (Fig 7). When the cranial endocast is oriented horizontally, the nasal cavity is more anteroventrally oriented in *Panoplosaurus* and more horizontal in *Pawpawsaurus*. The variation of the airway pattern observed in the nasal cavities of ankylosaurs has been related to variation on the production of sounds [13]. This is interesting because vocalizing vertebrates generally produce sound frequencies within the range of their hearing [62]. The more complex nasal cavity plus the enlarged lagena present in ankylosaurids such as *Euoplocephalus* [13, 17], versus the simpler nasal cavities plus shorter lagena in nodosaurids such as *Panoplosaurus* [13] and *Pawpawsaurus* may be related to the range of sounds these animals produced and received in a family level.

### Inner ear and hearing capabilities

The fluid-filled ducts within the three semicircular canals sense angular accelerations of the head and operate in conjunction with reflex arcs to cervical and extraoccular muscles to stabilize the head and maintain visual fixation [63]. In *Pawpawsaurus* the anterior semicircular canal is not markedly larger than the posterior semicircular canal, whereas in *Euoplocephalus* the anterior semicircular canal seems to be at least twice the size of the posterior semicircular canal ([13] see their Fig 8I). The inner ear of *Kunbarrasaurus ieversi* [9] is markedly different from that of *Euoplocephalus* and *Pawpawsaurus*, being proportionally enormous relative to the size of the skull, with short crus communis and short semicircular canals. Except for these three genera, there is no published information on ankylosaur labyrinth morphology. Comparative studies in extant mammals established the relationship between locomotor agility and canal size, indicating that sensitivity increases with increasing of canal size [64]. Moreover, recent studies [63] state that biped dinosaurs exhibit relatively larger canals. In sauropods, the enlargement of anterior semicircular canal in particular was associated with behavioral patterns that require agility in the head movements [41]. Within ankylosaurs, an increment of agility in the head movements in ankylosaurids when compared to nodosaurids can be suggested; however, the lack of inner ear data in most taxa prevents further interpretations at this point.

The length of the lagenar ducts represent the dimensions of the auditory sensory epithelium, which in turn are related to the hearing frequency sensitivity and auditory capability [65, 66]. The length of the lagena in *Pawpawsaurus* is approximately 4mm (Fig 4). According to Manley [65], this length of basilar membrane falls within that of the extant caiman, sensitive to a frequency of about 2–4 kHz. This result also is congruent with the maximum 3kHz level stated by Gleich et al. [67] for large dinosaurs. However, ankylosaurid dinosaurs (i.e., *Euoplocephalus* [13] and *Tarchia* [AP-C, pers. obs.]) show an extremely elongate lagena, possible the longest within dinosaurs. The length of the lagena correlates highly with hearing range [62], indicating not only that hearing was an important sense for ankylosaurids [13], but they also heard a wider range of frequencies than most dinosaurs. The relatively shorter lagena in *Pawpawsaurus* may indicate that taxon perceived a lower range of sounds than ankylosaurids such as *Euoplocephalus* [17] and *Tarchia* [19].

### Presence vs absence of floccular recess in ankylosaur endocrania

A remarkable trait is the presence of the flocculus of the cerebellum in the cranial endocast of some ankylosaurs ([13] see their Fig 8E,I) and [19], which is observed because it was large enough to leave an impression on the anterior side of the vestibular eminence (= floccular recess). In *Euoplocephalus* [13], *Tarchia* [19] and *Talarurus* [19, 20], the flocculus is not particularly large but it is well defined as a pyramidal projection posterodorsal to the roots of CNs V–VII on the lateral side of the endocast, slightly posteriorly projected towards the anterior semicircular canal of the inner ear. The flocculus is characteristic of theropods but is absent in most sauropods, with few exceptions such as the rebbachisaurids *Nigersaurus* [41] and MMCH-PV 63 [42], the dicraeosaurid *Dicraeoesaurus* [68] and the titanosauriformes *Giraffatitan* (“*Brachiosaurus*” in [46]). The development of the flocculus in non-avian theropod dinosaurs has been historically associated with bipedalism [69], and consequently its presence was expected in bipedal ornithischians [70]. However, the floccular recess has not been documented in any other bipedal or quadruped ornithischian braincase including ornithopods (e.g., *Camptosaurus*: YPM 1856); hadrosaurs (e.g., *Anatosaurus*: YPM 2134), and ceratopsians (e.g., *Triceratops*: USNM V5740, ROM 59423, ROM 59424; *Pachyrhinosaurus*: [33]). Galton [71] illustrates a flocculus in the endocasts of *Stegosaurus ungulatus*, *S*. *stenops*, and *Kentrosaurus aethiopicus*, although the floccular recess is not present in another specimen of *Stegosaurus* sp. (YPM 1853, pers. obs).

Within ankylosaurs, the flocculus (or more specifically the floccular recess) was first identified in the ankylosaurid *Euoplocephalus* [17], and more recently using CT scans, in *Tarchia* [19] and *Talarurus* [19]. The flocculus leaves no impression in the cranial endocasts of the nodosaurids *Pawpawsaurus*, *Struthiosaurus* (YPM 57173, [11], and an unnamed specimen from Japan ([15] see their Fig 4A). It is also absent in the basal ankylosaur *Kunbarrasaurus ieversi* [9].

As mentioned, the development of the flocculus in quadrupedal dinosaurs is rare [41, 42, 46, 68], and its paleobiological implications are poorly understood [72]. Recent studies on bird brain evolution state that the flocculus functions are not simply related to flight capabilities [73] but principally with gaze stabilization capabilities [74]. Within ankylosaurs flocculus (or its impression on the vestibular eminence, the floccular recess) has been identified only in ankylosaurids so far. Based on the statement of Walsh et al. [74] (that the development of the flocculus is related to an increase of gaze stabilization), we hypothesize that the development of the flocculus plus an enlarged anterior semicircular canal in ankylosaurids, is related to a higher gaze stabilization capability when compared to nodosaurid ankylosaurs. In this context, ankylosaurids are the only family of ankylosaurs bearing a heavy tail club [6], a structure probably evolved for delivering forceful impacts through the powerful swing of the tail [75, 76]. Although swinging behavior of the tail has been tested positively in ankylosaurids, it remains unknown whether the active movements of the club tail were used for intraspecific combat or interspecific defense [74]. The hypothesized higher gaze stabilization in ankylosaurids (based on the neuroanatomy), may have been related to the particular head movements demanded by the active use of the heavy club-tail.

## Conclusions

The endocranial cast of *Pawpawsaurus* described here is one of the most complete nodosaurid endocasts known; comparisons with other ankylosaurs suggest that the reduction of the flocculus is characteristic of nodosaurids. The first complete inner ear morphology is also described for this group. The orbitocerebral vein, the rostral middle cerebral vein and the dorsal head vein were identified for the first time in *Pawapawsaurus*. The position of the foramina for cranial nerves II–VI and the internal carotid artery were re-interpreted, whereas the nasolacrimal canal and the dorsal alveolar canal were described using CT scans.

The new information on the endocranial morphology of *Pawpawsaurus* add more anatomical data that has potential use not only in taxonomy and phylogeny, but also in the paleobiology of this group of dinosaurs through interpretations based on the relative development of sense organs, particularly olfaction, hearing and balance. For example, in extant vertebrates it has been shown that the endosseous labyrinth closely follows the path and shape of the membranous semicircular ducts [77], and also that the length of the lagena correlates highly with hearing range [62]. In *Pawpawsaurus* and also in the basal ankylosaur *Kunbarrasaurus ieversi* [9] the length of the lagena indicates that these taxa heard a lower range of frequencies than Late Cretaceous ankylosaurids such as *Euoplocephalus* [13] and *Tarchia* [19]. Finally, the olfactory region of the nasal cavity in *Pawpawsaurus* is relatively smaller than that present in the Late Cretaceous nodosaurid *Panoplosaurus* and the ankylosaurid *Euoplocephalus*, suggesting a less acute sense of smell for the Early Cretaceous form. However, when compared to theropod dinosaurs, the olfactory ratios of these three ankylosaur taxa are similar to that present in ceratosaurs, being only overcome by the ratios present in allosauroids and tyrannosaurids [28] (see their Table 1). The morphology of the rostral and caudal loops of the airway path in *Pawpawsaurus* and *Panoplosaurus* indicates greater ossification of the internal laminae and lateralization of the anterior airway loop in Late Cretaceous *Panoplosaurus* compared to Early Cretaceous *Pawpawsaurus*.

## Supporting Information

S1 File 3D pdf of brain and nasal cavities of *Pawpawsawurus campbelli* (FWMSH93B.00026). Using Adobe Reader, click on figure to activate 3D functionality. Skull, cranial endocast, nasal cavities and neurovascular passages related to the nasal cavity can be turned off and turned on individually. Skull can be turned semitransparent to allow observation of endocranial features.
(PDF)

S2 File 3D pdf of braincase, brain and inner ear of *Pawpawsawurus campbelli* (FWMSH93B.00026). Using Adobe Reader, click on figure to activate 3D functionality. Skull, isolate braincase, cranial endocast and inner ear can be turned off and turned on individually. Skull can be turned semitransparent to allow observation of endocranial features.
(PDF)
